# *Clitoria ternatea* L. (Butterfly Pea) Flower Against Endometrial Pain: Integrating Preliminary In Vivo and In Vitro Experimentations Supported by Network Pharmacology, Molecular Docking, and Molecular Dynamics Simulation Studies

**DOI:** 10.3390/life14111473

**Published:** 2024-11-13

**Authors:** Najneen Ahmed, Nazifa Tabassum, Parisa Tamannur Rashid, Basrat Jahan Deea, Fahmida Tasnim Richi, Anshuman Chandra, Shilpi Agarwal, Saima Mollick, Kaushik Zaman Dipto, Sadia Afrin Mim, Safaet Alam

**Affiliations:** 1 Department of Pharmacy, East West University, Dhaka 1212, Bangladesh; nazifa.tabassum@ewubd.edu (N.T.); parisa.rashid@ewubd.edu (P.T.R.); basrat.deea@ewubd.edu (B.J.D.); 2019-1-70-026@std.ewubd.edu (K.Z.D.); 2020-1-70-005@std.ewubd.edu (S.A.M.); 2 Department of Pharmaceutical Chemistry, Faculty of Pharmacy, University of Dhaka, Dhaka 1000, Bangladesh; tasnim.fahmida.du@gmail.com; 3 School of Physical Sciences, Jawaharlal Nehru University, New Delhi 110067, India; anshuman2022@jnu.ac.in (A.C.); shilpiagarwal@mail.jnu.ac.in (S.A.); 4 Pharmaceutical Research Division, BCSIR Dhaka Laboratories, Bangladesh Council of Scientific and Industrial Research (BCSIR), Dhaka 1205, Bangladesh; rsaimamollick@gmail.com; 5 Chemical Research Division, BCSIR Dhaka Laboratories, Bangladesh Council of Scientific and Industrial Research (BCSIR), Dhaka 1205, Bangladesh

**Keywords:** *Clitoria ternatea*, GO pathway, KEGG pathway, network pharmacology, molecular docking, molecular dynamics simulation, ADMET, anti-inflammatory, analgesic, endometriosis

## Abstract

*Clitoria ternatea* L. (CT) is a perennial herbaceous plant with deep blue flowers native to tropical Asia. This work explores the endometrial pain (EP) regulation of CT flower through a multifaceted approach. Phytochemical screening unveiled the presence of alkaloids, steroids, flavonoids, glycosides, and tannins in CT flower methanolic extract (ME). In the in vitro membrane stabilizing experiment, the ME demonstrated 91.47% suppression of heat-induced hemolysis. Upon carrageenan-induced paw edema assay conducted on male Swiss albino mice at doses of 200 mg/kg and 400 mg/kg, 65.28% and 81.89% inhibition rates, respectively, of paw edema were reported. For the same doses, upon acetic acid-induced-writhing assay, 75.6% and 76.78% inhibition rates, respectively, were observed. For network pharmacology analyses, a protein–protein interaction network was constructed for 92 overlapping gene targets of CT and EP, followed by GO and KEGG pathway enrichment analyses. Network pharmacology-based investigation identified the anti-EP activity of CT to be mostly regulated by the proteins SRC homology, ESR1, and PI3KR1. Physicochemical, pharmacokinetic, and toxicity property predictions for the compounds with stable ligand–target interactions and a molecular dynamics simulation for the highest interacting complex further validated these findings. This work affirmed the anti-EP role of CT flower against EP, suggesting a probable molecular mechanism involved.

## 1. Introduction

Endometriosis is a debilitating gynecological disease affecting 10–15% of women worldwide [1]. It is often termed as an estrogen-dependent disorder that is associated with the growth of endometrial glands and stroma in regions other than the uterus [2]. Processes like proinflammatory, proangiogenic, endocrine, and immune activities play key roles in the overall prognosis of the disease [3]. Recently, the homeobox (HOX) gene, which plays a key role in the development of the female reproductive system and is responsible for the regulation of several transcription factors involved in the differentiation and positioning of cells during embryogenesis, has been considered the key player behind the development of the disease [4]. The chronic nature of endometrial pain and other symptoms can lead to crucial psychological suffering, including anxiety, depression, and a sense of isolation, adversely affecting quality of life. In addition, endometrial pain and symptoms frequently result in decreased productivity and absenteeism from work, and impacted women may lose 10.8 h of work per week on average [5]. As a result, endometriosis may have long-term financial effects on women, such as decreased lifetime wages and retirement savings, which may exacerbate gender differences in financial well-being [6].

Currently, medications such as progestogen, NSAIDS, GnRH antagonists (GnRH-As), selective estrogen receptor modulators (SERMs), combined oral contraceptives, aromatase inhibitors (AIs), novel products like resveratrol, IL-1 antagonists, prostaglandin E2 (PGE2) receptor antagonists, and dopamine receptor antagonists [7] are used against endometriosis. GnRH-As appear to be the most promising medication in endometriosis treatment [8]. The treatment plan may include surgical alternatives such as laparoscopy, laparotomy, or hysterectomy, which involves removal of the ovaries. The existing medicinal and surgical therapies for endometriosis have a fairly high recurrence rate. Additionally, approved endometriosis medications might cause melancholy, insomnia, and bone loss [9]. Due to problems with the existing therapeutic approaches, individuals are beginning to favor herbal medications because of their mild nature and health benefits [10].

**Figure 1:**
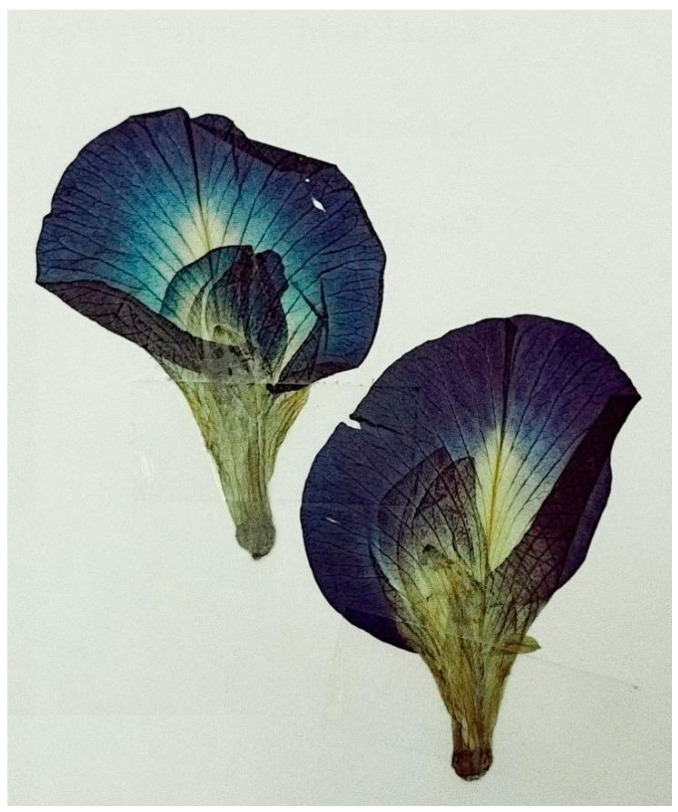
Dried flower of *C. ternetea* L.

*Clitorea ternatea* is a member of plant family Fabaceae and is a herbaceous perennial flowering plant commonly known as Butterfly pea (Figure 1). Although its origins are unknown, this plant is endemic to tropical Asia and Africa. It is currently found in Madagascar, South America, India, Southern and Eastern Africa, and the Western Indian Ocean [11]. The Butterfly pea flower is unique for its blue-hued petals, which have been widely utilized in Southeast Asian cuisine as a natural food-coloring agent [12]. Butterfly pea flower tea has been popularized as blue tea for its easy availability, affordability, and anti-inflammatory and antioxidant qualities, which can help relieve inflammation and chronic pain [13]. Owing to these diverse pharmacological effects, *Clitorea ternatea* has been traditionally used as a neuroprotective agent, an antimicrobial agent, an anti-inflammatory agent, and an anti-pyretic agent, making it a well-accepted remedy in treating anxiety, infection, pain, and inflammation. Moreover, Butterfly pea also promotes digestion, weight loss, diuresis, glaucoma alleviation, and blood glucose control. Butterfly pea flower is rich in polyphenolic constituents such as kaempferol, anthocyanins, phenolic acid, myricetin, and quercetin. Moreover, lipophilic constituents, including fatty acids such as palmitic acid, linolic acid, and arachidic acid, phytosterols such as campesterol, sitostanol, and sigmasterol, and various tocols like α-tocopherol and γ-tocopherol, are also present in the flowers of Butterfly pea [14]. These constituents can provide the medicinal effects of blue pea flowers, establishing their status as functional foods [15]. Due to the presence of promising phytochemicals, blue tea has the potential to alleviate pain, inflammation, and menstruation difficulties [16].

Traditional herbal medicines have been utilized for their therapeutic benefits for centuries, yet understanding their pharmacological effects remains challenging due to the complex interactions among multiple bioactive compounds. In response to this challenge, network pharmacology, a relatively newer computational field, has transpired as a propitious methodology to develop an understanding of the holistic mechanisms underlying herbal remedies [17]. First introduced by Hopkins in 2008, network pharmacology offers a paradigm shift by emphasizing the “multi-compounds, multi-target, and multi-disease” model of drug action, in contrast to the traditional “a-drug, a-gene, a-disease” framework [18]. Through the construction of network models integrating molecular, cellular, and organism-level interactions, an overall understanding of the intricate correlations between drugs, targets, and diseases can be achieved using network pharmacology [19]. The molecular docking approach can further validate bioactive compound and hub-gene-expressed critical protein interactions [20].

Previously, *Pueraria lobata*, known by the native name ‘kudzu’, has been found to have aromatase inhibitory activity, which has been proven to be very effective in the treatment of endometriosis [21]. Similarly, the hexane fraction of black aged garlic (*Allium sativum)* has been found to have inhibitory effects on tumor necrosis factor (TNF)-activated endometriotic stromal cells [22]. *Uncaria tementosa* extract, *Silybum marianum* extract, and *Calligonum comosum* extracts have been reported to be effective in the treatment of endometriosis [23]. An extensive literature survey indicates that prostaglandin E2, cyclooxygenase-2, NFκB, and estrogen cytokines are key players in the prognosis of the disease; thus, their receptors are considered potential targets for relieving endometriosis [24,25,26].

This study explored the prospective effects and molecular mechanism of an 80% methanolic crude extract of *C. ternatea* flower extract in alleviating endometriosis and related pain through network pharmacology and molecular docking-based analysis of the target proteins and active phytoconstituents followed by a molecular dynamics simulation analysis for the ligan–protein complexes [27]. We also investigated the anti-inflammatory and analgesic activities of the crude extract of *C. ternatea* flower through a range of in vitro and in vivo experiments.

## 2. Materials and Methods

### 2.1. Plant Material and Reagents

The reagents and chemicals used in the study included methanol (Sigma Aldrich Ltd., Tokyo, Japan), sodium chloride (50 mM), disodium hydrogen phosphate, and sodium dihydrogen phosphate to prepare phosphate buffer and freshly drawn human blood for erythrocyte (RBC) suspension. Analytical-grade reagents and solvents were purchased from a local chemical supplier (Active Fine Chemicals Ltd., Dhaka, Bangladesh) and used in this study.

Aceclofenac was used as a standard for evaluating the anti-inflammatory and antinociceptive effects of the crude methanolic extract. For this purpose, raw Aceclofenac was obtained from Incepta Pharmaceuticals Ltd. (Dhaka, Bangladesh). Carrageenan and acetic acid were used to induce paw edema and analgesia, respectively. They were procured from Sigma Aldrich (St. Louis, MO, USA).

The plant was obtained from the indoor facilities of Bailey Road, Bangladesh (latitude N 23°44.4524′, longitude-E 90°24.1467′). Khondokar Kamrul Islam, scientific officer, National Herbarium Bangladesh, Mirpur, Dhaka, identified and authenticated the plant sample, which was retained at the National Herbarium and Department of Pharmacy, East West University, Bangladesh, under the accession number DACB-94816 for future reference. The sample was sent to a herbarium directly after plucking and prior to collecting on a large scale. Upon confirmation, it was obtained in higher quantities and subjected to shade drying.

### 2.2. C. ternatea Flower Extract Preparation

After collection, shade-dried flowers of *C. ternatea* were powdered to obtain 240.5 g and were macerated with 3.0 L of 80% methanol for 45 days with occasional shaking. Afterward, with the help of a cotton plug followed by Whatman filter paper, the mixture was filtered and subjected to a rotary evaporator (Heidolph, Saffron Walden, UK), maintaining low-pressure conditions at 40 °C to acquire a concentrated crude methanolic extract, followed by rotary evaporation; to ensure complete removal of the solvent, the concentrated crude extract was left on a fume hood in the presence of a desiccant (SiO_2_) overnight (extraction efficiency 39.9%, 400 mg/gm dry weight of flower extract).

### 2.3. Phytochemical Screening

The phytochemical analysis of *C. ternatea* was carried out using previously described protocols [28]. The Molish test was performed to identify carbohydrates. Alkaloid identification was conducted by using Mayer’s reagent, Hager’s reagent, Wagner’s reagent, and Dragendroff’s reagent. Flavonoid identification utilized concentrated HCl, and 5% FeCl_3_ was added to the crude extract in the presence of distilled water. The presence of blue-black coloration or precipitation confirmed the presence of tannins. Steroid identification was performed by adding chloroform followed by concentrated H_2_SO_4_. The presence of red color in the chloroform layer ensures the presence of steroids.

### 2.4. Experimental Animals

Swiss albino male mice (body weight: 20–25 gm, average weight 23.2 ± 0.6 gm) aged 6 weeks were purchased from an animal resource facility, the International Center for Diarrheal Disease Research, Bangladesh (ICDDR, B). Housing was carried out at the animal house of the Department of Pharmacy, East West University. Standard polycarbonate cages were used to house the mice. ICDDR, B formulated food was fed to the mice. The temperature and humidity of the room were maintained at 25 ± 1 °C and 60 ± 5%, respectively, and the research protocol was approved with a 12 h dark/light cycle. Approval for the research protocol was obtained the Animal Ethics Committee of the State University of Bangladesh, Dhaka (2023-01-04/SUB/A-ERC/004), before initiating the study. Upon commencement of the study, the animals were subjected to euthanasia using intra-peritoneal administration of a 150 mg/kg dose of pentobarbital. Strict maintenance of the protocols of the Care and Use of Laboratory Animals of the National Institute of Health and the ARRIVE (Animal Research Reporting in vivo Experiments) guidelines were followed throughout the study.

Human *(Homo sapiens)* subjects were involved in the in vitro evaluation of membrane-stabilizing activity, for which healthy individuals with no known medical history of each sex were chosen. Blood was collected at the medical center of East West University under the direct supervision of a professional nurse. Informed consent was obtained from all subjects involved in the study. Approval for the research protocol was obtained from the Animal Ethics Committee of the State University of Bangladesh, Dhaka (2023-01-04/SUB/A-ERC/004), before initiating the study.

### 2.5. Membrane-Stabilizing Activity

This study involves the investigation of the membrane-stabilizing properties of the sample solution exposed to heat by involving techniques leveraging the Shimadzu UV spectrophotometer (Shimadzu, Kyoto, Japan). The absorbance of the supernatants was measured at 540 nm [19]. This method acts as an in vitro means to identify whether the test sample possesses anti-inflammatory activity or not [29].

#### Hemolysis Induced by Heat

In two sets of centrifuge tubes, about 2 mg/mL of extract, 30 μL of erythrocyte suspension, and 5 mL of isotonic buffer were taken. A similar preparation was made for two additional sets but excluding the extract. After gently swirling the tubes, one set was cooled in an ice bath at 0–5 °C while the other set was heated at 54 °C in a water bath for 20 min. Afterward, the formed supernatant was diluted, and its absorbance was evaluated at 540 nm. The percentage of inhibition or the rate of hemolysis acceleration was determined by employing the following equation:

Percent (%) inhibition of hemolysis = [{1 − (OD2-OD1)}/(OD3 − OD1)] × 100%(1)

where OD1 = absorbance of unheated test sample, OD2 = absorbance of heated test sample, and OD3 = absorbance of heated control sample.

### 2.6. Evaluation of Anti-Inflammatory Activity

The carrageenan-induced paw edema method was employed to assess in vivo anti-inflammatory potential of the crude methanolic extract of *C. ternatea* flower [30]. Here, normal saline was used as control and the crude methanolic extract was given at 200 mg/kg and 400 mg/kg b.w doses. Aceclofenac was given as standard by dissolving it in normal saline using tween 80 as a solubilizing agent (dose 25 mg/kg b.w) [31]. All four groups contained five mice each. Control, test, and standard mice were fed orally, and percentage inhibition of paw edema was measured at the end of the 1st, 2nd, 3rd, and 4th hours, followed by subcutaneous injection of carrageenan in the right hind paw of the test mice.

### 2.7. Evaluation of Peripheral Analgesic Activity

The acetic acid-induced-writhing method was employed to evaluate the peripheral analgesic activity of the test samples [32]. Crude methanolic extract of *C. ternatea* flower (200 mg/kg and 400 mg/kg b.w doses) and standard Aceclofenac (25 mg/kg b.w) were given orally to the test animals [33]. Normal saline was used as control to compare and nullify the effects of any residual solvent. Standard, control, and two test groups consisted of five test animals each. Acetic acid was injected intra-peritoneally to induce pain in the form of writhing. Percentage inhibition of writhing for test and standard was evaluated and compared.

### 2.8. Network Pharmacology-Based Analysis

#### 2.8.1. Collection of Constituents and Targets for *C. ternatea*

The Indian Medicinal Plants, Phytochemistry and Therapeutics (IMPPAT 2.0) database (retrieved from https://cb.imsc.res.in/imppat/ (accessed on 2 February 2024)) was used for the initial listing of the phytoconstituents, and eleven phytoconstituents were found to be present in the *C. ternatea* flower petals [34]. Additionally, an extensive literature search was carried out using scholarly databases such as PubMed and web search engines such as Google Scholar, using the keywords “Phytoconstituents of *Clitoria ternatea* flower”, “compound isolated from Butterfly pea flower”, and “bioactive molecules separated from *C. ternatea* flower”. Extensive literature searches contributed to the enrichment of the phytoconstituent list. Overall, fifty-nine compounds were reported to be present in the *C. ternatea* flower. Further screening of the listed constituents was performed based on the bioavailability score and drug-likeness properties using the swissADME online tool (retrieved from http://www.swissadme.ch/ (accessed on 2 February 2024) [35]). Compounds with a high bioavailability score (≥0.5) and not violating Lipinski’s rule of five were selected for further analysis.

Subsequently, the SwissTargetPrediction (retrieved from http://www.swisstargetprediction.ch/predict.php/ (accessed on 22 February 2024)) web database was searched to identify potential targets of the compounds [36]. The compound target genes were selected based on probability score. Target genes having probability values equal to or greater than 0.2 were selected. The targeted genes of the studied phytochemicals were then subjected to removal of redundancy and preparation of an exclusive gene list.

#### 2.8.2. Collection of Targets for Endometriosis, Inflammation and Endometrial Pain

Gene targets for endometriosis, inflammatory pain, and inflammation were collected using two databases—GeneCards (retrieved from https://www.genecards.org/ (accessed on 22 February 2024)) and DisGeNET (retrieved from https://www.disgenet.org/ (accessed on 22 February 2024)) [37,38]. Keywords like endometriosis, inflammatory pain, and inflammation were used to search putative genes related to the selected maladies. For the genes obtained from DisGeNET, a Gene–Disease Association score (sGDA) of 0.2 was chosen as the cutoff score. The gene list obtained from GeneCards was refined by selecting genes with a relevance score higher than or equal to 50%. Gene redundancies were removed from these two lists, and a final list of exclusive genes for endometrial pain was prepared.

The compound target genes (Section 2.4) of *C. ternatea* were matched with the disease target genes of concern through Venny 2.1 (retrieved from https://csbg.cnb.csic.es/BioinfoGP/venny.html (accessed on 22 February 2024)) for intersection analysis, and the genes common for both disease and compounds were then considered as prospective target genes for further analysis [39].

#### 2.8.3. Analysis of Common Targets for Endometriosis

The common gene targets were imported to the STRING platform (retrieved from https://string-db.org/ (accessed on 22 February 2024)) where protein–protein interaction was studied [40]. *Homo sapiens* was chosen as the species, and a high confidence score (>0.9) was set as the minimum confidence threshold. The resultant protein–protein interaction (PPI) was then visualized using Cytoscape 3.10.1 [41]. Cytohubba plugin was used to identify the core targets based on node connection degree [42].

#### 2.8.4. GO and KEGG Pathway Enrichment Analysis by Target Genes

Shared gene targets of the isolated compounds and the studied diseases were analyzed using the Database for Annotation Visualization and Integrated Discovery ((DAVID) (retrieved from https://david.ncifcrf.gov (accessed on 25 February 2024)) for Gene Ontology (GO) analysis and Kyoto Encyclopedia of Genes and Genomes (KEGG) pathway enrichment analyses [43]. The official gene symbols of the shared target genes were imported into the DAVID web server, and *Homo sapiens* was selected as the species of interest. For the GO enrichment analysis, the “Functional Annotation Tool” was utilized, and the GO functions were annotated using the terms biological process (BP), cellular components (CCs), and molecular functions (MFs), and the respective charts were downloaded and processed. The KEGG pathway chart was downloaded from the “Annotation Result Summary”. The obtained GO and KEGG enrichment analysis charts were rearranged by filtering the results with a p-value less than 0.05 and sorted from the largest to smallest enrichment scores in terms of −log10 (*p*-value) and fold enrichment, respectively. The top ten results of the GO enrichment analysis and top 20 KEGG pathway enrichment analyses were graphed and visualized with bar diagrams and bubble diagrams, respectively, with the help of the SR Plot web server (retrieved from https://www.bioinformatics.com.cn/srplot (accessed on 25 February 2024)).

### 2.9. Molecular Docking

Three-dimensional structures of the selected ligands were searched and collected using the PubChem database (https://pubchem.ncbi.nlm.nih.gov/ (accessed on 22 February 2024)) [44]. To compare the binding capacity of the selected phytochemicals, elagolix (PubChem Id-11250647), which is an FDA-approved drug for relieving endometriosis, was chosen. The structures were saved in SDF format, and energy minimization was performed using PyRx [45]. RCSB Protein Data Bank (retrieved from https://www.rcsb.org/ (accessed on 22 February 2024)) was used for structure retrieval of the top three hub targets i.e., SRC (PDB ID: 2H8H), ESR1 (PDB ID: 3ERT), and PIK3R1 (PDB ID: 5XGJ), using pdb format. Protein preparation was performed by removing co-crystallized water and ligands, followed by adding hydrogen. Energy minimization of the target proteins was performed using SwissPDBviewer (version 4.1) [46]. The active site of the proteins was identified using a literature survey and utilizing exiting co-crystallized ligand coordinates. The Autodock Vina (version 0.8) plugin from PyRx was used to study molecular docking interactions between the target protein’s active site and selected ligands [47]. Visualization of the ligand–target interactions was achieved using Biovia Discovery Studio version 2021 [48].

### 2.10. In Silico ADMET Analysis

The absorption, distribution, metabolism, excretion and toxicity (ADMET) profile of the selected phytoconstituents based on the molecular docking score was studied using the online server pkCSM (https://biosig.lab.uq.edu.au/pkcsm/ (accessed on 22 February 2024)) [49]. Preliminary pharmacokinetic and toxicity data generated by the server help to identify potential lead compounds in terms of safety and druggability.

### 2.11. Molecular Dynamics Simulation

**Figure 2:**
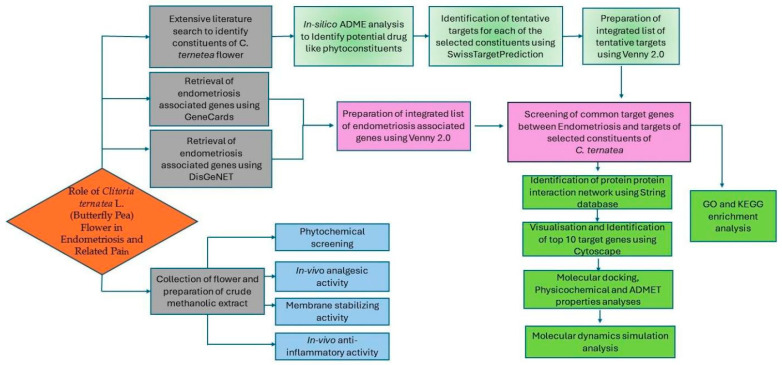
Workflow diagram for the identification of drug-like phytoconstituents of *Clitoria ternatea* flower against endometrial pain using network pharmacology analysis followed by in vitro, in vivo, and in silico validation.

Molecular dynamics (MD) simulation was performed for the top protein (SRC, PDB ID: 2H8H) and ligand complex showing the highest docking interaction score. The Schrodinger Desmond package (Desmond Molecular Dynamics System version 1.9.1, D. E. Shaw Research, New York, NY, USA, 2021) was utilized to analyze the movements of the proteins docked with ligands and the outcomes were compared to the apo form, generated by the MD simulation. As a force field, OPLS2005 was applied while a simple point charge model was used for the task. All the analyzed target–ligand complexes were neutralized by adding counter ions (47 Na^+^ and 44 Cl^−^), and to simulate the human physiological system, a 0.15M Na^+^ level was maintained. The prepared complexes underwent relaxation through the Desmond default protocol [50]. The NpT ensemble was set at 300 K temperature and 1 bar of pressure for the MD simulation run for 100 ns. Following the completion of the simulation, the results of the ligand–target complexes were acquired as RMSD, RMSF, and ligand RMSF. Figure 2 demonstrates the methodology adopted in the present study.

### 2.12. Statistical Analysis

The data obtained from biological evaluation were subjected to statistical analysis using one-way analysis of variance (ANOVA) followed by Dunnett’s multiple comparison test. All the observed values were expressed as mean ± SEM (n = 5). Data for which *p* values were found as *** *p* < 0.001 and ** *p* < 0.01 were considered statistically significant as compared to the control group. The analysis was carried out using Microsoft Excel (version 2010) and GraphPad version 10.2.2.

## 3. Results

### 3.1. Phytochemical Screening

Phytochemical analysis of CT exposed the presence of alkaloids, flavonoids, glycosides, steroids, and tannins (Table 1).

### 3.2. Evaluation of Membrane-Stabilizing Property

As the lysosomal membrane and the membrane of red blood cells resemble one another, the effects of medications that stabilize the erythrocyte membrane might have a similar impact on cells responsible for releasing chemicals that can cause inflammation. Therefore, they may be capable of showing anti-inflammatory effects. The capacity of the crude methanolic extract of *C. ternatea* flower to stabilize membranes in vitro was studied under heat-induced conditions.

**Figure 3:**
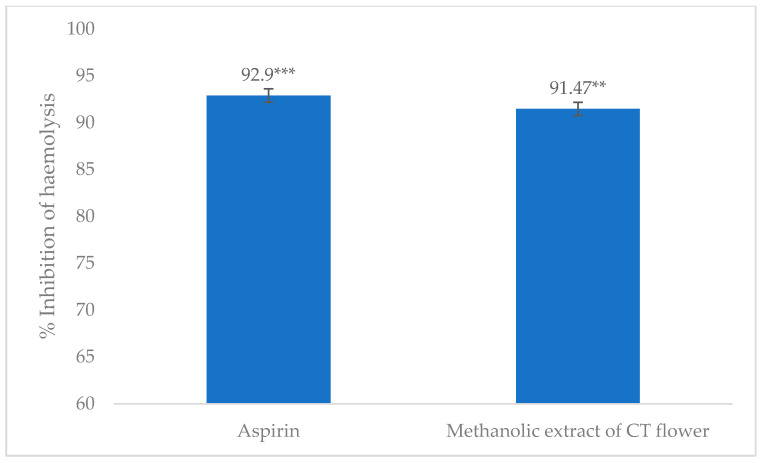
Effect of crude methanolic extract of *C. ternetea* flower on heat-induced hemolysis of normal RBC. Here, *** *p* < 0.001 and ** *p* < 0.01.

The absorbance for the determination of the anti-inflammatory activity of the crude methanolic extract of *C. ternatea* flower in heat-induced conditions is summarized in Table 2. Each test was repeated three times for better precision. Percentage inhibition of hemolysis by the standard and test sample is shown in Figure 3.

This study revealed that methanolic flower extract of *C. ternatea* demonstrated significant inhibition of hemolysis of RBCs in heat-induced conditions, having 91.47% inhibition of hemolysis in comparison to 92.90% inhibition by the standard aspirin (0.10 mg/mL). Based on these results, it can clearly be stated that the sample under experimentation might have the ability to stabilize the RBC membrane and, by extension, the membranes of the inflammation-causing lysosomal cells, thereby providing a potential anti-inflammatory effect.

### 3.3. Evaluation of In Vivo Anti-Inflammatory Activity

**Figure 4:**
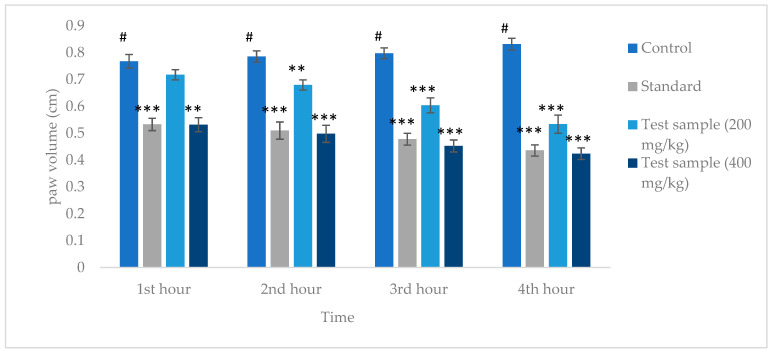
Effects of *C. ternatea* flower methanolic extract and Aceclofenac on paw diameter after carrageenan injection. Each value represents the mean ± SEM (n = 5), *** *p* < 0.001 and ** *p* < 0.01, compared with control (one-way ANOVA followed by Dunnet’s multiple comparison test). # indicates control, against which comparison was made.

The carrageenan-induced paw edema test induces acute inflammation in test animals. The mean paw volume in each test group for four consecutive hours upon administration of carrageenan is shown in Figure 4. The control group (normal saline) showed a gradual increase in paw volume with time, whereas both standard and test samples showed a gradual decrease in paw volume over the four-hour test period. All the test data were found to be statistically significant except for a 200 mg/kg dose of the test sample right after the first hour of treatment. Moreover, no adverse effects were observed among test animals, thus ensuring safety of the prepared extract.

The standard Aceclofenac showed 52.79%, 63.22%, 70.56%, and 80.38% inhibition of paw edema. In contrast, the crude extract at the 200 mg/ kg dose showed 21.03%, 31.81%, 48.80%, and 65.28%, and at the 400 mg/kg, dose it showed 56.22%, 64.885, 74.09%, and 84.89% inhibition of paw edema of at the end of the first, second, third, and fourth hours, respectively (Table 3).

### 3.4. Evaluation of Peripheral Analgesic Activity

The peripheral analgesic activity of the crude methanolic extract of *C. ternatea* flower was evaluated using the acetic acid-induced-writhing method, where writhing times were the evaluation index. Aceclofenac at a dose of 25 mg/kg b.w. showed 77.49% inhibition, whereas methanolic extract showed 75.6% and 76.68% inhibition of writhing at a dose of 200 mg/kg and 400 mg/kg, respectively, when compared to the control group (Table 4). No signs of adverse effects were evident among test animals, which ensured the safety of the prepared extract.

### 3.5. Collection of Plant Active Constituents and Target Genes

In total, 59 different bioactive constituents were identified (Supplementary File) following an extensive literature search, from which 18 were selected as they showed no more than one violation of Lipinski’s rule of five [11,15,16,34,51]. The selected constituents were also predicted to have a high bioavailability score (Table 5).

All of these compounds were previously identified to be present in the *C. ternatea* flower through a number of spectrophotometric and other identification methods. Using SwissTargetPrediction (http://www.swissadme.ch/ (accessed on 2 February 2024)), an exclusive list of 279 gene targets of these eighteen compounds were identified.

### 3.6. Collection of Disease-Related Genes and Putative Target Identification

**Figure 5:**
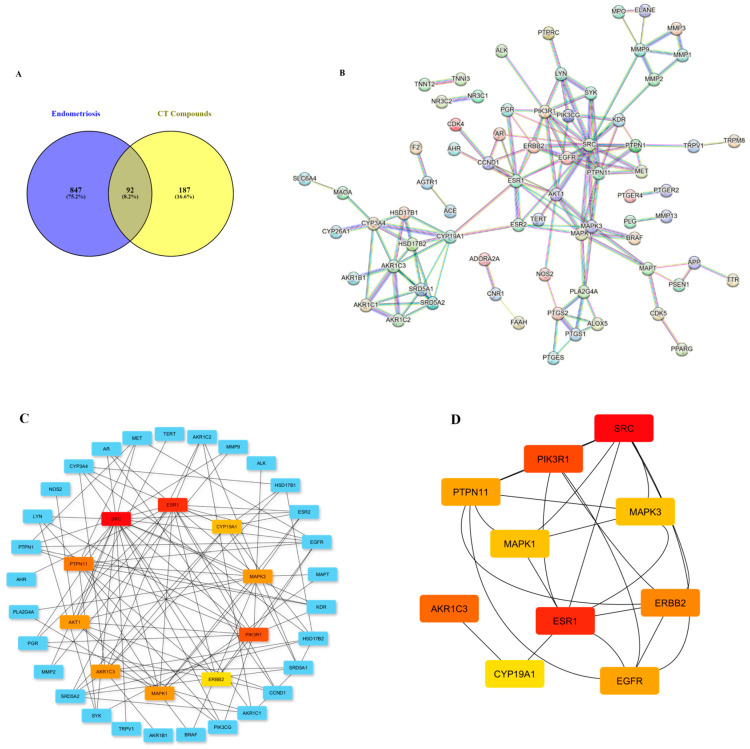
Schematic representation of construction of protein–protein interaction (PPI) network. (**A**) Venn diagram of the intersection of target genes of the phytoconstituents and the disease-specific genes. (**B**) PPI network preparation for 92 putative target genes using STRING database. (**C**) Top ten hub target genes with extended network neighbors. (**D**) Top hub genes within themselves. The darker the red hue, the more connected and important the gene within the disease–compound network.

Using the two databases, i.e., GeneCards and DisGeNET, 734 and 341 genes related to endometriosis and related pain were retrieved. Upon compilation, a total of 939 genes were obtained; 92 common target genes were finally identified by the interaction of disease-related genes and selected constituents of the *C. ternatea* flower (Figure 5A). These target genes were subjected to further analysis.

### 3.7. Protein–Protein Interaction Analysis

The common 92 target genes were analyzed for the purpose of constructing a protein–protein interaction (PPI) network. For better scrutiny, a minimum confidence score of 0.9 was selected and disconnected nodes were hidden (Figure 5B).

The resultant network was then saved as a tab-separated value (tsv) file and visualized using Cytoscape 3.10.1. Using the CytoHubba (version 0.1) plugin, the top ten hub targets were identified based on node connection degree. The top 10 chosen proteins were SRC (non-receptor tyrosine kinases), ESR1 (estrogen receptor 1), PIK3R1 (phosphatidylinositol 3-kinase regulatory subunit 1), PTPN11 (protein tyrosine phosphatase non-receptor type 11), AKR1C3 (Aldo-keto reductase), AKT1 (alpha serine/threonine protein kinase 1), MAPK1 (mitogen-activated protein kinase 1), MAPK3 (mitogen-activated protein kinase 3), CYP19A1, and CYP3A4 (cytochrome p450 enzymes) (Figure 5C,D). The deep red color represents the interconnection of the particular protein within the network. (Figure 5C,D). The greater the node degree, the higher the probability of the anti-endometriosis effect through targeting that protein. Table 6 lists other parameters for screening the node proteins from the PPI network.

Based on the degree, the three most important node proteins in the PPI network, i.e., SRC, ESR1, and PIK3R1, were selected for molecular docking analysis.

### 3.8. GO and KEGG Pathway Enrichment

**Figure 6:**
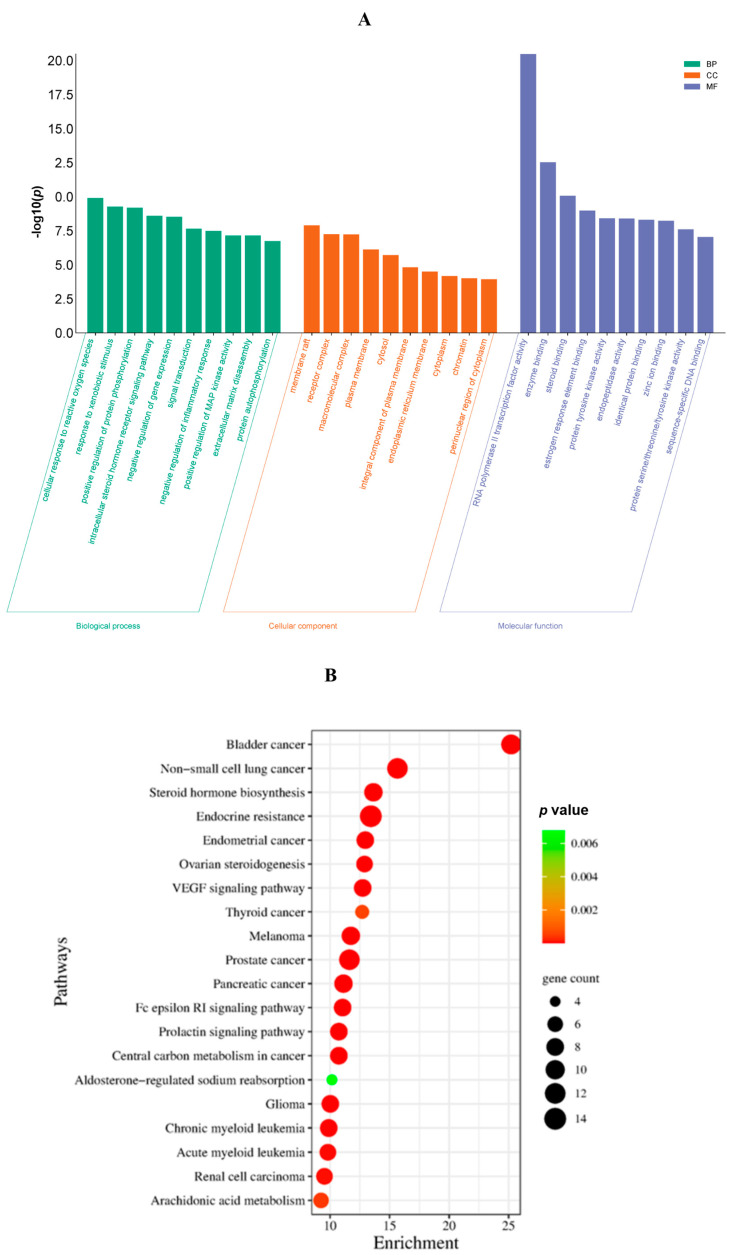
(**A**) GO functional and (**B**) KEGG pathway enrichment analyses.

The GO functional enrichment and KEGG pathway enrichment analyses for the compound–disease network of 92 shared target genes were carried out using the DAVID and SR Plot web servers. The functional annotation of the GO enrichment analysis included analysis of the enrichment of the genes for biological process (BP), cellular components (CCs), and molecular functions (MFs). The top ten biological functions with a p-value less than 0.05 and the largest –log10 (*p*-value) were selected and plotted in Figure 6A. The most significant BP was the cellular response to reactive oxygen species involving the genes MMP2, MMP3, AKR1C3, MAPK1, AKT1, MAPT, MMP9, EGFR, and MAPK3. The most significantly enriched cellular component was the membrane raft, which is enriched with the genes LYN, APP, PTPRC, CNR1, SRC, KDR, TEK, MAPT, PSEN1, TRPM8, EGFR, and SLC6A4. The top MF enriched with the studied genes is the RNA polymerase II transcription factor activity.

Using *p <* 0.05 as the screening condition, 106 enriched signaling pathways were screened. The signaling pathways, which were enriched mainly by the common genes of *C. ternatea* flower phytoconstituents and endometrial pain, were found to include bladder cancer, non-small-cell lung cancer, steroid hormone biosynthesis, endocrine resistance, endometrial cancer, and ovarian steroidogenesis, among others. SRD5A2, SRD5A1, HSD17B1, AKR1C1, HSD17B2, AKR1C3, AKR1C2, CYP3A4, and CYP19A1 were significantly enriched in steroid hormone biosynthesis, and CCND1, ERBB2, MAPK1, AKT1, BRAF, PIK3R1, EGFR, and MAPK3 were found to enhance the endometrial cancer pathway (Figure 6B).

### 3.9. Molecular Docking

Eighteen selected ligands were docked against the top three proteins, i.e., SRC kinase or non-receptor tyrosine kinase (PDB 2H8H), ESR1 or estrogen receptor 1 (PDB 3ERT), and PI3K or phosphatidylinositol 3-kinase (PDB 5XGJ), obtained from protein–protein interaction. From the analysis, it was found that flavylium, kaempferol, and quercetin exhibited overall promising binding affinity against all three targets, suggesting their probable association with endometrial pain relief. Elagolix, an FDA-approved gonadotropin-releasing hormone (GnRH) antagonist used for the treatment of endometriosis, was also docked against the selected targets to compare the binding affinities of the phytoconstituents. Binding affinities are shown in Table 7.

**Figure 7:**
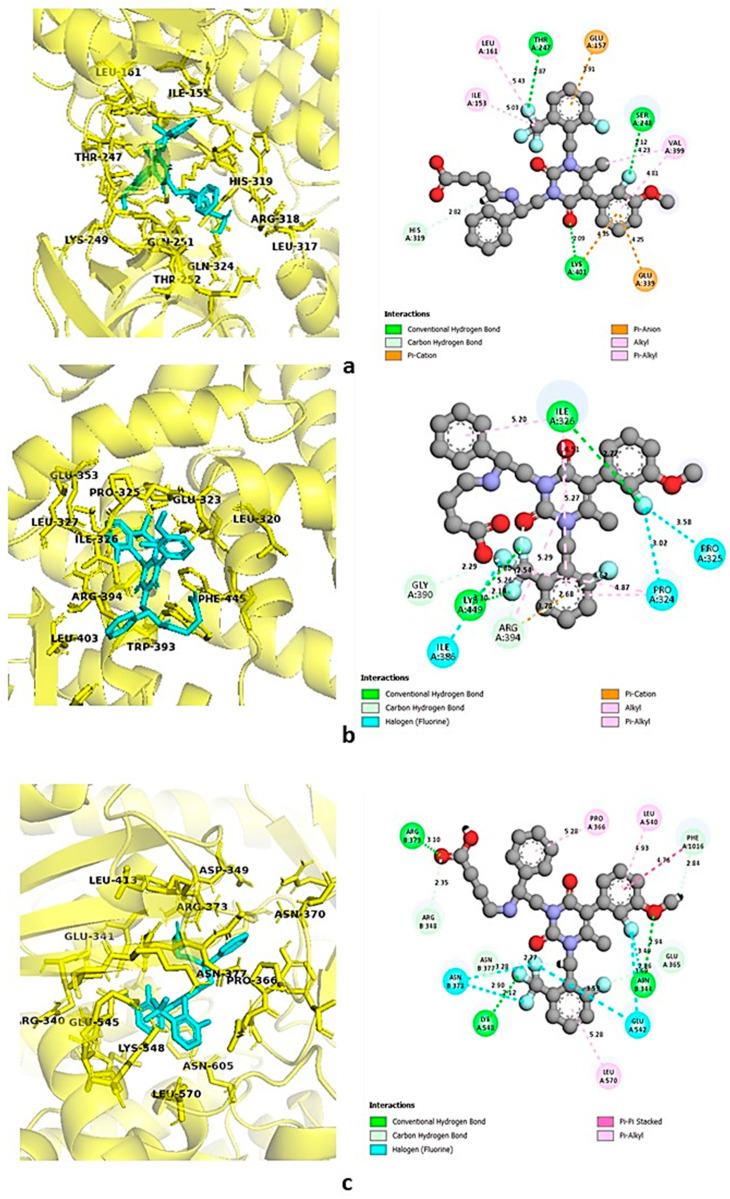
Binding interaction of standard elagolix with (**a**) SRC, (**b**) ESR1, and (**c**) PIK3R1. Green: conventional hydrogen bond; pink-violet: hydrophobic; orange: pi-cation/pi-anion/pi-sulfur; cyan: carbon–hydrogen bond.

**Figure 8:**
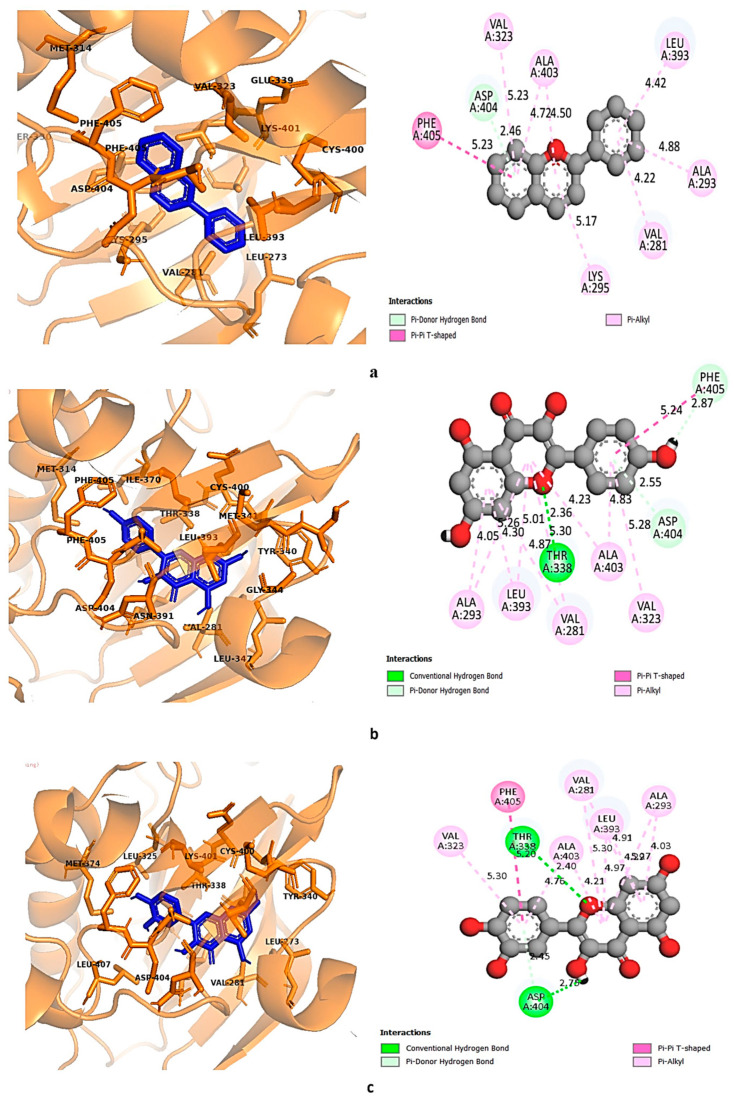
Binding interaction of SRC with (**a**) flavylium, (**b**) kaempferol, and (**c**) quercetin. Green: conventional hydrogen bond; pink-violet: hydrophobic; orange: pi-cation/pi-anion/pi-sulfur; cyan: carbon–hydrogen bond.

**Figure 9:**
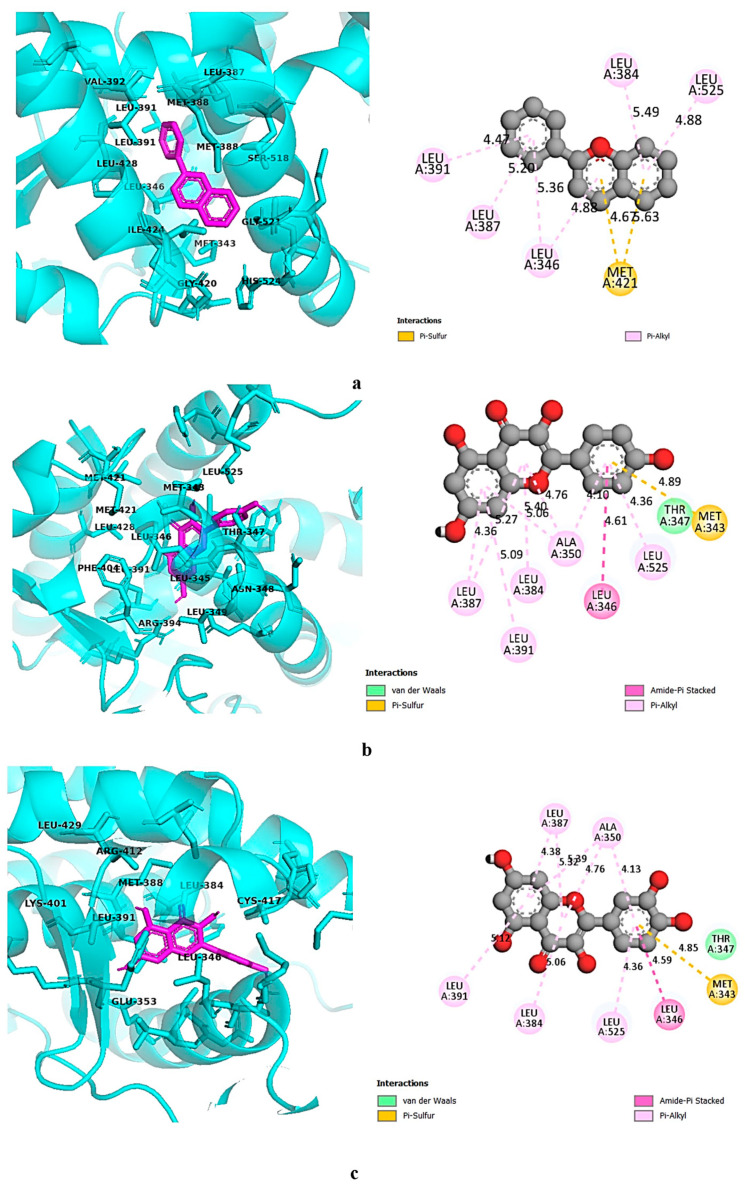
Binding interaction of ESR1 with (**a**) flavylium, (**b**) kaempferol, and (**c**) quercetin. Green: conventional hydrogen bond; pink-violet: hydrophobic; orange: pi-cation/pi-anion/pi-sulfur; cyan: carbon–hydrogen bond.

**Figure 10:**
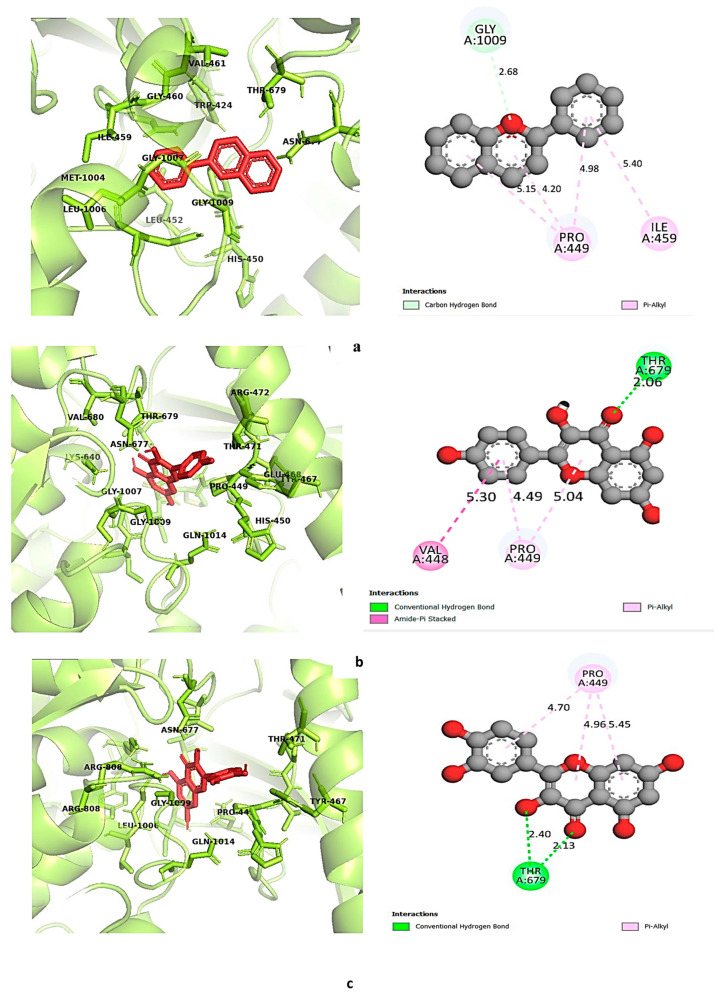
Binding interaction of PIK3R1 with (**a**) flavylium, (**b**) kaempferol, and (**c**) quercetin. Green: conventional hydrogen bond; pink-violet: hydrophobic; orange: pi-cation/pi-anion/pi-sulfur; cyan: carbon–hydrogen bond.

Both kaempferol and quercetin were found to exhibit strong affinities towards all three selected targets; the binding affinity of kaempferol ranged between −8 kcal/mol and −8.8 kcal/mol, and binding affinities of quercetin ranged between −7.8 kcal/mol and −8.8 kcal/mol. Although flavylium showed the highest affinity towards SRC, which was −9.1 kcal/mol, its binding affinities towards PIK3A and ESR1 were lower than those of kaempferol and quercetin. All three selected ligands had higher binding affinities towards SRC and ESR1 than the standard drug elagolix. On the contrary, elagolix showed the highest affinity towards PIK3R1 compared to the selected ligands. For a better understanding of the ligand–protein interaction, both three-dimensional and two-dimensional figures with color-coded binding interaction types were generated by Discovery Studio version 2021 [57] and are demonstrated in Figure 7, Figure 8, Figure 9 and Figure 10.

### 3.10. In Silico ADMET Analysis

In silico prediction of the absorption, distribution, metabolism, excretion, and toxicity (ADMET) parameters for the compounds with the best-docked scores, flavylium, kaempferol, and quercetin, was performed using pkCSM (Table 8). This predictive analysis will provide a better perception of and insight into their pharmacokinetic properties as well as their toxicity to the human body [58].

All the analyzed compounds were predicted to show moderate intestinal absorption (>70%) and moderate (flavylium) to poor (kaempferol, quercetin) BBB permeability, with all being predicted as a P-gp substrate. All the compounds were anticipated to be CYP1A2 metabolic enzyme inhibitors. From toxicity prediction, none of the analyzed compounds were found to be toxic for AMES, hERG I and II inhibition, and hepatotoxicity checkpoints.

### 3.11. Molecular Dynamics Simulation for Selected Protein–Ligand Complexes

**Figure 11:**
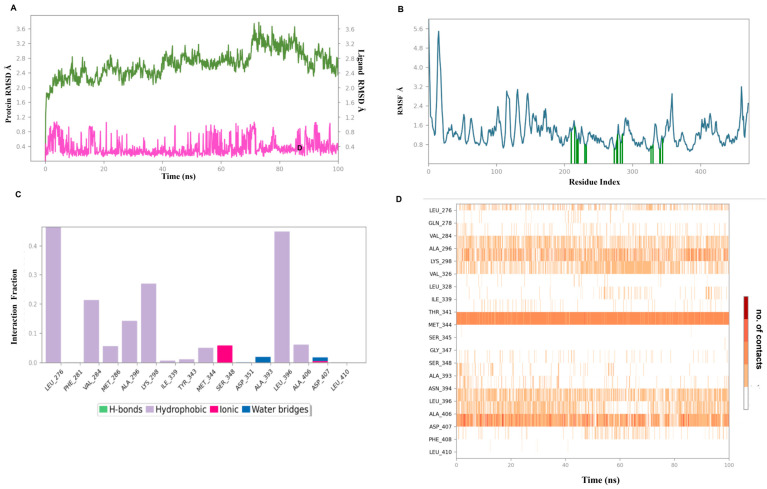
(**A**) RMSD evaluation (green for SRC homology, purple for flavylium). (**B**) Protein RMSF assessment for the docked complex of flavylium and SRC homology (2H8H) in a 100 ns MD simulation. (**C**) Flavylium was observed throughout the MD run. Four types of interactions can be distinguished: water bridges (blue), hydrophobic (lavender), ionic (magenta), and H-bonds (green). The stacked bar indicates the percentage of interaction time in the whole 100 ns analysis period, where 0.4 indicates 40% of the time. (**D**) Timeline representation of protein–ligand contacts where a darker orange hue indicates several contacts of the same amino acid residue with the ligand.

The ligand–protein binding interaction was further validated through MD simulation studies. The ligand–target complex with the highest binding interaction scores, SRC homology–flavylium, was considered for MD simulation for 100 ns. Figure 11 depicts the MD simulation outcomes for the SRC homology and flavylium complex.

From the root mean square deviation (RMSD) analysis, moderate stability of the complex in the selected biological system was observed based on the low RMSD value for flavylium and SRC homology ranging from 0.2 to 1.0 Å and from 2.0 to 3.6 Å, respectively. Initially, the RMSD value of SRC homology was stable up to 70 ns, followed by a sharp hike up to 3.6 Å from 75 ns to 85 ns, which then finally stabilized back to the initial RMSD value range. The root mean square fluctuation (RMSF) value for SRC homology was in an acceptable range with a few peaks at the loop region. Protein residues around 250 and 350 were found to possess RMSF values below 2 Å, depicting the stable interacting regions of the protein with flavylium. The protein–ligand interaction analysis from the MD simulation and molecular docking matched as none of the residues was found to form hydrogen bonds. The strongest interaction pattern was hydrophobic interaction with LEU276, LYS298, and LEU398, which were maintained well throughout the MD simulation period. Flavylium also showed an ionic interaction with SER346 for a short duration. From the MD simulation results, it could be inferred that flavylium may act as a potential compound against SRC homology protein in lowering endometrial pain.

## 4. Discussion

The present study investigates the phytochemical composition of crude methanolic extract of *C. ternetea* flower followed by an evaluation of its analgesic and anti-inflammatory potential, which was confirmed by in vitro and in vivo analysis. Later, network pharmacology-, in silico-, and molecular dynamic simulation-based approaches validated the molecular basis of using *C. ternetea* flower extract in the treatment of endometrial pain. Three molecular targets, i.e., SRC homology, PIK3R1, and ESR1, were selected based on a network pharmacology-based approach, and good binding affinity was obtained for three out of eighteen of the selected ligands. Molecular dynamic simulation validated the stability of the flavylium–SRC complex, which gave the best score in docking analysis.

Women of reproductive age are particularly vulnerable to endometriosis, with no curative or preventive measures, adversely affecting daily life [59]. Blue tea or *C. ternatea* flower tea is one of the popular herbal tea preparations that claim to relieve endometriosis-related pain by reducing oxidative stress and inflammation by scavenging the detrimental radicals [60]. *C. ternatea* flower’s phytochemicals’ antioxidant activities can reduce the synthesis of inflammatory mediators and enzymes, which could aid in the fight against inflammation [61]. In this study, preliminary phytochemical analysis of the crude methanolic extract of the *C. ternatea* flower revealed the presence of phytochemicals of diversified classes, including glycosides, alkaloids, flavonoids, steroids, and tannins, which is similar to the findings of phytochemical analysis performed on the *C. ternatea* flower of Sri Lankan origin, which supported the antioxidant activity of the flower extract owing to these important classes of natural products [62].

Molecules with the ability to stabilize the erythrocyte membrane, owing to their similarities, may also stabilize the lysosomal membrane, potentially demonstrating anti-inflammatory effects by limiting the activity and release of cellular mediators [63,64]. The present investigation on the *C. ternatea* flower extract (2.0 mg/mL) demonstrated a potent anti-inflammatory effect as it inhibited 91.47% of hemolysis of the erythrocytes in comparison to 92.9% inhibition of hemolysis by the standard, aspirin (0.1 mg/mL). Similarly, in vivo studies unveiled promising anti-inflammatory activity in terms of the 400 mg/kg dose, almost similar to the activity given by the standard, Aceclofenac. The presence of ternatin anthocyanins, quercetins [65], kaempferol [12], and quercetin phytochemicals, like anthocyanins, kaempferol, quercetin, and myricetin glycosides, is responsible for the diversified pharmacological effects of Butterfly pea flower, including, anti-inflammatory properties, and therefore determines the impact of such extract on reducing symptoms associated with inflammation caused by toxins, trauma, or infections [51]. However, a study in Sri Lanka with aqueous extracts of *C. ternatea* roots stated that the anti-inflammatory activity of this plant might not be mediated via membrane stabilization, as different concentrations of the extract against standard aspirin showed no significant outcome [66].

In terms of peripheral analgesic activity, at both 200 mg/kg and 400 mg/kg doses, crude methanolic extract exhibited promising activity with 75.6% and 76.7% inhibition of writhing, which is similar to the analgesic activities reported for *C. ternatea* root and leaf crude extracts [67,68]. Several studies conducted in animal models have shown the analgesic potential of phytoconstituents like kaempferol, quercetin glycosides, and ellagic acid, all of which are also present in leaves of *C. ternatea* [69,70,71]. Thus, these chemicals may also be responsible for the analgesic effects of the crude methanolic extract of the flower of this species.

Network pharmacology has extensively been utilized to identify the anti-endometriosis mechanisms and related targets of a wide range of plant secondary metabolites [72,73,74]. In our study, we identified 939 targets of the eighteen orally bioavailable (OB ≥ 50%) drug-like compounds reported to be present in the *C. ternatea* flower and 279 disease targets for endometriosis. Intersection analysis revealed 92 common targets between the screened phytochemicals and endometriosis, suggesting the potential activity of these compounds against endometriosis by targeting these proteins [75]. Further protein–protein interaction analysis for the shared genes was carried out based on degree of centrality. Within the PPI network, target proteins with higher node degrees are considered the most centralized hub proteins [76]. Our analysis identified SRC, ESR1, PIK3R1, PTPN11, AKR1C3, AKT1, MAPK1, MAPK3, CYP19A1, and CYP3A4 as the ten most involved node proteins within the PPI network.

To validate the involvement of these node proteins as the targets of the screened compounds against endometriosis, three proteins with the topmost node degrees, SRC, PIK3R1, and ESR1, were selected for molecular docking study. Previously, evidence of proteins related to SRC kinase was found to be associated with endometriosis [77,78]. Involvement of the protein PI3K, as well as its mutations in the prognosis of endometriosis and associated cancers, has been reported in several studies [79]. A previous study involving bioinformatics-based analysis of the genes associated with endometriosis reported the involvement of PIK3R1 as a potential driver of the disease [80]. The role of estrogen in the pathophysiology of inflammation and endometriosis has been extensively reported in several studies [81,82]. The development of endometriosis is associated with several hallmarks, including estrogen dependence, inflammation, and progesterone resistance [83]. Thus, compounds with the potential to treat inflammation and having an affinity for estrogen receptors are good candidates for treating endometriosis.

Eighteen screened constituents were subjected to molecular docking interaction analysis, and flavylium, kaempferol, and quercetin showed promising binding affinities against all three protein targets. Standard elagolix, which is FDA-approved for treating endometriosis, exhibited good binding affinities against all three targets, and the result was comparable to the obtained values for kaempferol, flavylium, and quercetin. The antioxidant and anti-inflammatory activities, as well as the preventive role of both kaempferol and quercetin against fibrosis associated with the development of endometriosis, have been established by several studies [84,85,86]. Flavylium, an anthocyanin flavonoid, was found to be associated with anti-inflammatory activity, with specific affinity towards the ESR1 and ESR2 receptors [87]. Considering the involvement of hub genes and the impact of phytoconstituents in endometriosis, in silico studies along with PPI network analysis provide an understanding of the probable mechanism responsible for the use of Butterfly pea extract against endometriosis [88].

The results generated by molecular docking studies were further validated by MD simulation. Protein stability, stability upon binding of proteins with ligands, as well as conformational changes in protein upon ligand binding are studied under molecular dynamic simulation [89]. Usually, the root mean square deviation (RMSD) value between 2.0 and 3.0 Å indicates stable protein–ligand interactions over a 100 ns simulation period. Our studied complex, SRC–flavylium, exhibited RMSD values within the given range, although no stable value was seen for more than 30 ns. Running the simulation over a 200 ns period may provide a better understanding of the ligand–protein binding behavior [90]. In addition, the RMSF analysis hinted at the flexibility of the protein–ligand interaction, which was further supported by the interaction pattern analysis as there was no hydrogen bond present between the ligand and protein residues. Conventional hydrogen bonding provides stable ligand–target interaction [91,92], which justifies the presence of the protein RMSD and RMSF fluctuations for the SRC–flavylium complex. In silico physicochemical and ADMET property analyses did not predict any crucial violation of the drug-likeness properties and the possibility of severe adverse effects or toxicity, which assures the higher probability of success of the studied phytochemicals in the preclinical setting [93].

The GO functional annotation analysis categorized the common genes targeted by *C. ternatea* against endometrial pain under the terms “biological process”, “cellular process”, and “molecular functions”, with the -log10 (*p*-value) indicating the significance level of each term. The biological processes are the most significant, with processes such as “cellular response to stress”, “response to oxidative stress”, and “inflammatory response” being highly represented. These target gene-enriched biological processes suggest that *C. ternatea* may modulate cellular and systemic stress responses, potentially reducing the inflammation associated with endometrial pain [94]. In the category of cellular components, terms like “membrane raft”, “membrane micro-domain”, and “membrane region” were enriched significantly, indicating the involvement of *C. ternatea* in modifying the cellular membrane dynamics, which could influence cell signaling pathways related to pain perception [95]. The molecular function category features terms such as “protein binding”, “ion binding”, and “enzyme regulator activity”, indicating that the action of *C. ternatea* at the molecular level may involve modulation of enzymatic activities and ion channel functions, which are crucial in the transmission of pain signals [96].

The KEGG pathway enrichment analysis highlights critical pathways potentially involved in endometrial pain and influenced by the selected phytochemicals in *C. ternatea*. Steroid hormone biosynthesis plays an important role in endometrial function, and estrogens, the female sex hormone that is produced through this pathway, can exacerbate endometrial pain by promoting inflammation and angiogenesis, the two key factors in the pathogenesis of endometriosis [97]. Moreover, endocrine resistance, such as progesterone resistance, contributes to endometrial pain through estrogen dominance, which can stimulate inflammatory pathways and increase endometriosis symptoms, mostly endometrial pain [98]. Ovarian steroidogenesis is closely linked with endometrial changes, and dysregulation can contribute to conditions causing pain [99]. Each pathway offers a distinct mechanism potentially contributing to endometrial pain, illustrating the complex interplay of hormonal and angiogenic factors in endometrial pathophysiology.

## 5. Conclusions

Through network pharmacology-, molecular docking-, and dynamics-based analysis, as well as physicochemical and ADMET property prediction, our study tried to connect the outcomes of the preliminary in vitro anti-inflammatory and in vitro and in vivo analgesic activities to the molecular mechanisms involved in relieving endometrial pain by the drug-like constituents present in the *C. ternatea* flower. Our study reported a crucial connection with protein targets such as SRC, ESR1, and PIK3 in endometriosis. These target proteins were found to be involved in different mechanisms involved with endometrial prognosis and to be effectively targeted by quercetin, kaempferol, and flavylium present in *C. ternatea* flower. The flavylium–SRC homology interaction analysis from MD simulation provided insights into the binding behavior of flavylium with the protein. In silico physicochemical and pharmacokinetic property prediction for these three phytochemicals shed light on their possible success in further preclinical studies. This study provides a strong background and warrants further investigation involving distinct in vivo animal disease model studies as well as immune-blotting analysis to analyze the role of these key phytochemicals on the studied protein expression cascades.

## 6. Limitations

This study could not adopt advanced spectrophotometric approaches for the isolation, identification, characterization, and quantification of the phytochemicals present in the *C. ternatea* flower due to funding constraints. This study performed the preliminary pharmacological evaluation of the analgesic and anti-inflammatory activities of *C. ternatea* flower. The study plan was designed based on a number of previously reported articles, which included network pharmacology-based investigations followed by experimentation on required animal models, typical for generalized pain and inflammation without isolating the active constituents, utilizing literature reviews or available medicinal plant-based databases [100,101,102]. For better approximation of the experimental binding energy, rigorous binding free energy calculations through the linear interaction energy (LIE) method can provide further insights into docking stability [103]. As no specialized animal model for endometrial pain was utilized for the study, the results of network pharmacology and in silico and animal studies should be repeated specifically for endometrial pain in specialized models in the future to establish the mechanism of action of *Clitoria ternatea* flower extract in the treatment of the concerned ailment. Due to funding restrictions, this study only incorporated the experimental binding energies for the ligand–target complexes using open access software and tools.

## Figures and Tables

**Table 1:** Phytochemical screening of the crude methanolic extract of *C. ternatea* flower.

Phytochemicals	Observation
Alkaloids	+
Carbohydrates	−
Flavonoids	+
Glycosides	+
Saponins	−
Steroids	+
Tannins	+

(+) = present, (−) = absent.

**Table 2:** Absorbance of heat-induced erythrocyte suspension upon treatment with control, standard and test sample at 540 nm.

Sample	Concentration (mg/mL)	OD1	OD2	OD3
Control	-	-	-	1.09 ± 0.01
Aspirin	0.1	0.095 ± 0.000	0.171 ± 0.001	-
Methanolic extract of CT flower	2.0	0.082 ± 0.001	0.160 ± 0.000	-

OD = observed data; OD1 = absorbance of unheated test sample, OD2 = absorbance of heated test sample, and OD3 = absorbance of heated control sample. All data for OD1, OD2 and OD3 are expressed as mean ± S.E.M.

**Table 3:** Evaluation of anti-inflammatory activity of *C. ternatea* flower extract by measuring percentage inhibition of paw edema at different time intervals.

Group	% Paw Edema Inhibition			
	1st Hour	2nd Hour	3rd Hour	4th Hour
Control	No edema inhibition	No edema inhibition	No edema inhibition	No edema inhibition
Standard	52.79	63.22	70.56	80.38
Test sample (200 mg/kg)	21.03	31.81	48.80	65.28
Test sample (400 mg/kg)	56.22	64.88	74.19	81.89

**Table 4:** Evaluation of the analgesic activity of crude methanolic extract of *C. ternatea* flower using acetic acid-induced-writhing test.

Sample Code	Dose (mg/kg)	Number of Writhing Actions (Mean ± SEM)	% of Inhibition of Writhing
Control	0	84.4 ± 3.78	-
Standard	25	16.8 ± 2.52 ***	77.49
Test sample	200	20.6 ± 3.70 ***	75.60
	400	19.6 ± 3.262 ***	76.78

Each value represents the mean ± SEM (n = 5), *** *p* < 0.001, compared with control (one-way ANOVA followed by Dunnet’s test).

**Table 5:** List of phytochemicals reported to be present in the flower petal of *C. ternatea*.

Phytochemicals	PubChem ID	Predicted Oral Bioavailability Score	Method of Identification	References of Identification Method
Flavylium	145858	0.55	UV, NMR, LC-MS/MS	[52]
Kaempferol	5280863	0.55	UV, NMR, LC-MS/MS; LC-MS/Q-TOF	[52,53]
Quercetin	5280343	0.55	UV, NMR, LC-MS/MS; LC-MS/Q-TOF	[52,53]
Quercetion-3-o-β-d glucoside	5280804	0.55	UV, NMR, LC-MS/MS	[52]
Palmitic acid	985	0.85	HPLC-PDA, LC-MS/ESI	[54]
Stearic acid	5281	0.85	HPLC-PDA, LC-MS/ESI	[54]
Petroselinic acid	5281125	0.85	HPLC-PDA, LC-MS/ESI	[54]
Arachidic acid	10467	0.85	HPLC-PDA, LC-MS/ESI	[54]
Behenic acid	8215	0.85	HPLC-PDA, LC-MS/ESI	[54]
Phytanic acid	26840	0.85	HPLC-PDA, LC-MS/ESI	[54]
Phytosterols	12303662	0.55	HPLC-PDA, LC-MS/ESI	[54]
Campesterol	173183	0.55	HPLC-PDA, LC-MS/ESI	[54]
Stigmasterol	5280794	0.55	HPLC-PDA, LC-MS/ESI	[54]
Ellagic acid	5281855	0.55	HPLC-PDA, LC-MS/ESI	[54]
Caffeoylmalic acid	6124299	0.56	HPLC-PDA	[55]
β-Sitosterol	222284	0.55	HPLC-PDA, LC-MS/ESI	[54]
Sitostanol	241572	0.55	HPLC-PDA, LC-MS/ESI	[54]
Taraxerol	92097	0.55	HPTLC	[56]

Oral bioavailability: >0.50 is considered to be orally bioavailable; HPLC-PDA: high-performance liquid chromatography–photodiode array; LC-MS: liquid chromatograph–mass spectroscopy; Q-TOF: quadruple time of flight; ESI: electrospray ionization; UV: ultraviolet spectroscopy; NMR: nuclear magnetic resonance spectroscopy; HPTLC: high-performance thin-layer chromatography.

**Table 6:** The core targets of *C. ternatea* in the treatment of endometriosis and related pain.

Target Gene	Degree	Betweenness Centrality	Closeness Centrality
SRC	20	34.03333	1254.197
ESR1	13	31.16667	969.0654
PIK3R1	12	28.18333	213.9751
PTPN11	10	26.58333	95.60952
MAPK3	9	28.61667	540.8903
MAPK1	9	28.61667	540.8903
AKT1	9	27.95	343.9957
AKR1C3	9	22.15	179.1667
CYP19A1	8	25.6	1085.333
EGFR	7	25.05	30.84127

**Table 7:** Binding affinities (kcal/mol) of the selected ligands against the top three hub proteins.

Ligand	SRC (PDB ID 2H8H)		ESR1 (PDB ID 3ERT)		PIK3R1 (PDB ID 5XGJ)	
	Binding Energy (kcal/mol)	Residues Involved in H-Bonding	Binding Energy (kcal/mol)	Residues Involved in H-Bonding	Binding Energy (kcal/mol)	Residues Involved in H-Bonding
Flavylium	−9.1	-	−7.2	-	−7	THR 679A
Kaempferol	−8.8	THR 338A	−8	-	−8.1	THR 679A
Quercetin	−8.8	THR 338A, ASP 404A	−7.8	-	−8.2	THR 679A
Standard Elagolix	−8.6	THR 247A, SER 248A, LYS 401A	−5.9	ILE 326A, LYS 449A	−9.6	LYS 548A, ASN 344B, ARG 373B

RMSD was 0.0 for all the binding scores of Table 7.

**Table 8:** In silico prediction of pharmacokinetic and toxicity profile of flavylium, kaempferol, and quercetin reported to be present in *Clitoria ternetea* flower.

	Interpreted Value for Prediction	Flavylium	Kaempferol	Quercetin
**Absorption**				
Caco2 permeability (log Papp in 10^−6^ cm/s)	>0.90, permeable	1.631	0.032	−0.229
Human intestinal absorption (percentage)	<30%, poor absorption	96.182	74.29	77.207
Skin Permeability (log Kp)	>−2.5, low skin permeability	−2.128	−2.735	−2.735
P-glycoprotein substrate	Prediction based on built model	−	+	+
**Distribution**				
BBB permeability (logBB)	>0.3, permeable	0.454	−0.939	−1.098
VDss (human) Log L/kg	>0.45, high <−0.15, low	0.24	1.274	1.559
Fraction unbound	Prediction based on built model	0.147	0.178	0.206
**Metabolism**				
CYP2D6 substrate	Prediction based on built model	−	−	−
CYP3A4 substrate		+	−	−
CYP1A2 inhibitor		+	+	+
CYP2C19 inhibitor		+	−	−
CYP2C9 inhibitor		−	−	−
CYP2D6 inhibitor		−	−	−
CYP3A4 inhibitor		−	−	−
**Excretion**				
Total clearance (log mL/min/kg)	Prediction based on built model	0.716	0.477	0.407
Renal OCT2 substrate		−	−	−
**Toxicity**				
AMES toxicity	Prediction based on built model	−	−	−
Max. tolerated dose (MTD) (human) in log mg/kg/day	≤0.477, low MTD >0.477, high MTD	0.044	0.531	0.499
hERG I inhibitor	Prediction based on built model	−	−	−
hERG II inhibitor		−	−	−
Oral Rat Acute Toxicity (LD50) in mol/kg		1.848	2.449	2.471
Oral Rat Chronic Toxicity (LOAEL) in log mg/kg_bw/day		1.118	2.505	2.612
Hepatotoxicity		−	−	−

+ indicates yes and − indicates no; Caco-2 = human colorectal adenocarcinoma cells; BBB = blood–brain barrier; VDSS = steady-state volume of distribution; CYP = cytochrome P450; OCT2 = renal organic cationic transporter-2; hERG = human ether-go-go gene; LD = lethal dose; LOAEL = lowest observed adverse effect level. Prediction based on built model = graph signature model from the drug data set.

## Data Availability

All the data involved in this study are disclosed in the manuscript and throughout the Supplementary Files.

## Supplementary Materials

The supporting information can be downloaded at https://www.mdpi.com/article/10.3390/life14111473/s1, Table S1: Identifiers of the 59 compounds identified to be present in *C. ternatea* flower; Table S2. Identifiers and Screening criteria of the 18 screened compounds; Table S3. Exclusive list of the official gene symbols of the targets of the screened 18 compounds predicted by SwissTargetPredition; Table S4. Official gene symbol of the targets of Endometrial pain, endometriosis and inflammation retrieved from two databases; Table S5. Official gene symbol of the common genes targeted by both the 18 screened compound pool and the studied diseases; Table S6. GO analysis of the 92 common targets for 18 screened compounds and studied diseases (*p*-value < 0.05); Table S7. KEGG pathway enrichment analysis of the 92 common targets for 18 screened compounds and studied diseases (*p*-value < 0.05).
